# A qualitative study of hospital and community providers’ experiences with digitalization to facilitate hospital-to-home transitions during the COVID-19 pandemic

**DOI:** 10.1371/journal.pone.0272224

**Published:** 2022-08-18

**Authors:** Hardeep Singh, Carolyn Steele Gray, Michelle L. A. Nelson, Jason X. Nie, Rachel Thombs, Alana Armas, Christian Fortin, Hedieh Molla Ghanbari, Terence Tang

**Affiliations:** 1 Department of Occupational Science & Occupational Therapy, Faculty of Medicine, University of Toronto, Toronto, Ontario, Canada; 2 Bridgepoint Collaboratory for Research and Innovation, Lunenfeld-Tanenbaum Research Institute, Sinai Health System, Toronto, Ontario, Canada; 3 Institute of Health Policy, Management & Evaluation, Dalla Lana School of Public Health, University of Toronto, Toronto, Ontario, Canada; 4 Institute for Better Health, Trillium Health Partners, Mississauga, Ontario, Canada; 5 Hennick Bridgepoint Hospital, Sinai Health, Toronto, Canada; 6 Division of Physical Medicine and Rehabilitation, University of Toronto, Toronto, Canada; 7 Division of Hospital Medicine, Sinai Health System, Toronto, Canada; 8 Department of Family and Community Medicine, University of Toronto, Toronto, Canada; Public Library of Science, UNITED KINGDOM

## Abstract

**Background:**

The COVID-19 pandemic has triggered substantial changes to the healthcare context, including the rapid adoption of digital health to facilitate hospital-to-home transitions. This study aimed to: i) explore the experiences of hospital and community providers with delivering transitional care during the COVID-19 pandemic; ii) understand how rapid digitalization in healthcare has helped or hindered hospital-to-home transitions during the COVID-19 pandemic; and, iii) explore expectations of which elements of technology use may be sustained post-pandemic.

**Methods:**

Using a pragmatic qualitative descriptive approach, remote interviews with healthcare providers involved in hospital-to-home transitions in Ontario, Canada, were conducted. Interviews were analyzed using a team-based rapid qualitative analysis approach to generate timely results. Visual summary maps displaying key concepts/ideas were created for each interview and revised based on input from multiple team members. Maps that displayed similar concepts were then combined to create a final map, forming the themes and subthemes.

**Results:**

Sixteen healthcare providers participated, of which 11 worked in a hospital, and five worked in a community setting. COVID-19 was reported to have profoundly impacted healthcare providers, patients, and their caregivers and influenced the communication processes. There were several noted opportunities for technology to support transitions.

**Interpretation:**

Several challenges with technology use were highlighted, which could impact post-pandemic sustainability. However, the perceived opportunities for technology in supporting transitions indicate the need to investigate the optimal role of technology in the transition workflow.

## Introduction

The COVID-19 global pandemic declared on March 11, 2020, has led to unprecedented changes to the Canadian health system [1, 2]. Various measures were put in place to reduce the risk of coronavirus disease (COVID-19) transmission and prevent outbreaks, with restrictions added and removed depending on the pandemic progression [3]. For instance, health systems postponed or cancelled elective surgeries, restricted hospital visitor policies and cancelled numerous community services [4]. While hospital capacity in Ontario has historically been strained, there is an even more urgent need to discharge patients from the hospital to prevent overwhelming hospital systems with the management of surging COVID-19 admissions, in addition to already admitted patients [5–7].

One of the most transformational changes to the healthcare system during the COVID-19 pandemic has been the rapid uptake of digital health technologies [8, 9] to support continuous delivery of care while containing the spread of COVID-19 by minimizing physical interactions [9]. Digital health is broadly defined as the use of technologies, such as the telephone, mobile health (e.g. applications), health information technology (e.g. patient portals), personal computers (e.g. email) and the Internet (e.g. webpages, Youtube) to improve health [10, 11]. While some of these technologies existed long before the pandemic [9], implementation barriers had previously prevented their widespread uptake in clinical settings [12, 13]. During the global pandemic, regulatory changes, including data regulations and privacy policies, quickly removed many system-level barriers [9, 14]. In Ontario, the accelerated adoption of virtual care during the COVID-19 pandemic was also made possible due to the Ontario government’s investment and approval of billing codes and procedures to support telemedicine and virtual care in its commitment to modernizing care in the province [15]. Virtual care has helped keep patients, clinicians and families safe by reducing the potential for exposure to the virus and improving access to patient care [16].

Older adults are at a high risk of adverse health outcomes during hospital-to-home transitions [17–22]. Improving hospital-to-home transitions, particularly for older adults with complex care needs who transition more frequently due to higher care needs, is a high priority for health systems, including health service providers and researchers [23–27]. Coordinating hospital-to-home transitions is a complex task as multiple healthcare providers share the responsibility of care for a patient [28, 29] and older adults with complex care needs have higher service needs [26]. The challenges experienced by healthcare providers who provide transitional care have been well-documented in the pre-pandemic context [28, 30–32]. Some believe that the COVID-19 pandemic may catalyze changes within the health systems [3, 33–35]. In particular, this pandemic may be an opportunistic time to rethink transitional care processes [36]. The rapid adoption of technology may be used to address some of the pre-pandemic barriers to transitions, including poor communication and incomplete information transfers [37, 38]. Hospital and community providers are key players in facilitating hospital-to-home care transitions for older adults [28]. However, providers’ perspectives about how changes instituted in response to the pandemic have impacted care transitions for older adults remain understudied at the time of this study.

Given this disrupted healthcare context [1], we want to understand the experiences of healthcare providers who have been engaged in hospital-to-home transitions, specifically during the COVID-19 pandemic. A greater understanding of provider experiences during the COVID-19 pandemic can provide insight into positive changes to transitional care that may be sustained beyond the pandemic. Thus, we aimed to i) explore the experiences of hospital and community providers with delivering transitional care during the COVID-19 pandemic; ii) understand how rapid digitalization in healthcare helped or hindered hospital-to-home transitions during the COVID-19 pandemic; and iii) explore expectations of which elements of technology use may be sustained post-pandemic.

## Methods

### Design

A pragmatic qualitative descriptive study was conducted [39]. A pragmatic approach entails combining established qualitative approaches to apply to an implementation science study [40]. This approach was appropriate as pragmatic approaches are used to explore real-world situations [41] and it is aligned with an interpretivist paradigm [42].

### Ethics

The Research Ethics Boards of Trillium Health Partners (ID#1016) and Sinai Health System (MSH REB 20-0152-E) provided ethical approval for this study. We obtained informed verbal consent from all participants prior to data collection and documented the verbal consent process.

### Settings and participants

Based on the sample sizes anticipated to reach theoretical sufficiency in qualitative research using interviews (e.g. n = 9–17 [43]; n = 20–40 [44]) and our previous qualitative work, we aimed to recruit 20–30 hospital and community healthcare providers in this study. Individuals were eligible to participate in this study if they were: i) a regulated healthcare provider who ii) provided health services to older adults with complex care needs and iii) were involved in care transitions. The following types of healthcare providers were sought for this study: i) hospital providers working in acute medicine or rehabilitation units (e.g. physician, discharge planner/social worker, allied health, nurse), ii) primary care providers (e.g. physicians, nurses), and iii) home and community care providers (e.g. home care coordinators and clinician). Providers reflecting these perspectives were of interest as they share the responsibility of care for patients during care transitions [29, 45]. These providers typically work collaboratively to coordinate a safe hospital-to-home transition; for instance, hospital staff make referrals to home and community providers to manage a patient’s needs in the community, and primary care providers may follow-up on the patient’s non-acute care needs after discharge [46].

Hospital providers were recruited from two large healthcare organizations located in urban regions in Ontario, Canada. Site A, located in Toronto, Ontario, serves over 29,000 inpatients and 19,000 outpatient visits [47]. Site B, located in Mississauga, Ontario, has over 64,000 inpatient admissions and 276,000 ambulatory/outpatient visits [47]. Both organizations operate within a public healthcare system and provide general inpatient and outpatient medical care to patients across the health continuum. Community providers do not work for hospital organizations, but rather the regional home care agency. Hospital and community providers were recruited using snowball sampling techniques [48], wherein individuals within the networks of the research team were sent an email and recruitment flyer containing details of the study and asked to forward this information to eligible colleagues in their network (e.g. sharing the flyer with other staff on their respective hospital unit). Hospital providers were also recruited through the team’s network (e.g. the Digital Bridge Implementation Team at Site A and B) at the two Sites. For example, an Implementation Team Member identified potential participants and with the individual’s consent, shared their contact information with HS or JN. Community providers were recruited from the Implementation Team’s community health network, wherein the study flyer was shared by the Implementation Team with others within their network and then interested participants contacted a research team member who provided additional study details and confirmed their study eligibility. The sampling approaches aligned well with our goal to speak with individuals experiencing the phenomenon of interest and the need to generate rapid qualitative results during the pandemic [49].

### COVID-19 context

This study was conducted during Ontario’s second and third COVID-19 waves (January–April 2021) [50]. Since March 2020, the province of Ontario instituted various measures to control the spread of the pandemic, which were subject to alternations over time due to shifting evidence related to COVID-19 prevention and management [51–54]. For instance, public health encouraged residents to shelter in place (other than for essential reasons). During the pandemic, elective surgeries/procedures were postponed, hospitals and care home visitors were restricted [51–54] and hospitals across Ontario experienced periods of significant capacity pressures [6, 7, 52, 53]. Within primary care, patient visits had rapidly shifted from in-person to virtual care [55, 56]. Some services within the home and community care sector also shifted to virtual and faced staffing shortages during the COVID-19 pandemic [57, 58].

### Data collection

In line with a qualitative descriptive approach [39], 30 to 60-minute semi-structured interviews were conducted. Interview questions (see S1 Appendix) asked providers to reflect on experiences facilitating hospital-to-home transitions for older adults during the pandemic and how technology helped or hindered the transition process. For example, we asked questions such as ’Can you tell me about what the discharge process looks like today and how the discharge process is different from pre-COVID’ and ’How has information sharing/communication with patients, caregivers and other providers changed?’ Interviews were conducted remotely by phone or on Zoom Healthcare (a Personal Health Information Protection Act compliant videoconferencing platform). Interviews were led by HS (an occupational therapist and postdoctoral trainee with qualitative expertise) or JXN (a research coordinator with qualitative research experience). They were audio-only or audio-video recorded and the interviewers took field notes during the interviews.

### Data analysis

**Fig 1:**
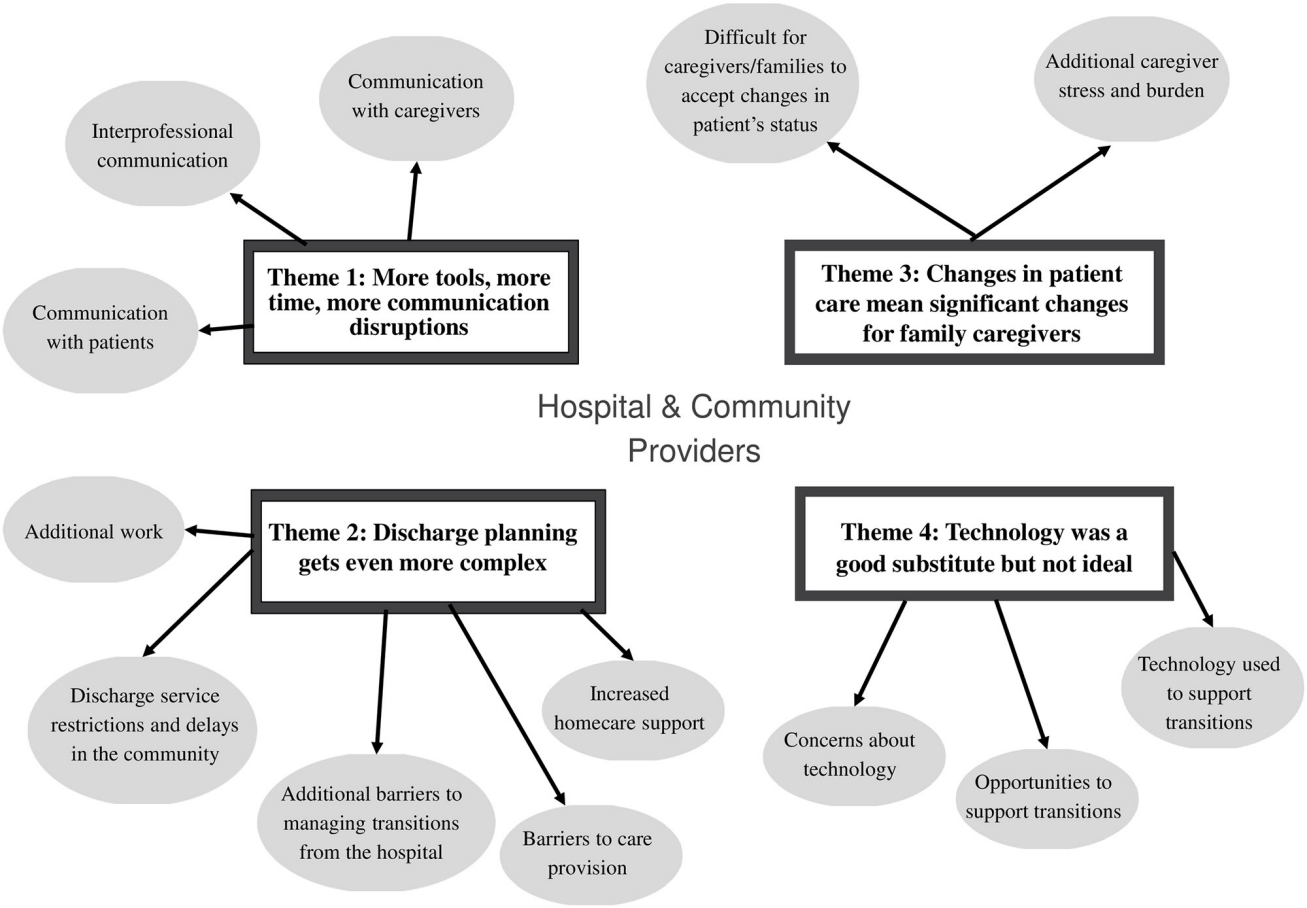
Summary of themes and subthemes.

A team-based rapid qualitative analysis approach [59] was used to analyze the interview data. A rapid analysis approach was appropriate here to generate "timely results in rapidly changing situations," given the rapidly evolving healthcare context during the COVID-19 pandemic [60]. Comprehensive details about this analysis approach have been reported in another publication [59]. The analysis began with two to three researchers independently listening to (audio-only interviews) or watching (for audio-video interviews) each of the anonymized interview recordings and taking field notes. The lead author (HS) created visual maps, which were visual diagrams of the key concepts/ideas, information and concepts [61] from each interview (i.e. ‘individual maps’) on a graphic design website or Microsoft Powerpoint. The maps were reviewed synchronously during regular team meetings held on Zoom or asynchronously and then modified based on input from a second and third team member (CSG: Scientist; RT: MSc Student/Research coordinator; AA: PhD Student/Research coordinator; JXN: Research coordinator; TT: Clinician Scientist), who have a variety of professional backgrounds (e.g. medicine, anthropology, health services, nursing, sociology), but whom all have qualitative research experience. While the lead analyst was involved in analyzing data from both Sites, we assigned different team members to analyze data from each Site to effectively capture Site-based differences (i.e. JXN and TT were involved in the analysis of data from providers recruited from Site A, while CSG, RT and AA were involved in the analysis of data from providers recruited from Site B). During their review, the team members looked for patterns between the different individual maps and offered suggestions for how the maps could be combined. Maps that were perceived by the research team to display similar data were combined to form ‘meta-maps’. For instance, we combined individual maps to form based on provider setting (i.e. hospital or community), discipline (i.e. physicians) and Site (i.e. Site A and B) and then reviewed the meta-maps to determine the best fit. After determining the best fit, the team further revised the best-fit meta-map to ensure it reflected the key themes; this analysis stage resulted in the ‘final map’ (see Fig 1). Multiple researchers conducted the analysis, which enhanced analytic rigour [59, 62–64]. Rigour was further enhanced through an audit trail of the analysis and following the Standards for Reporting Qualitative Research guidelines [65].

## Results

Of the 16 providers who participated in this study, 11 worked in a hospital (five were from Site A and six were from Site B), and five worked in a community setting (three were recruited through the Site A Implementation Team’s community network and two through the Site B Implementation Team’s community network). Of the 16 interviews, 15 were only audio-recorded, and one was audio and video recorded, and they ranged between 17:16–52:13 minutes in length. Provider roles included: primary care physician (n = 3), hospital-physician (n = 4), occupational therapist (n = 3), social worker (n = 1), physiotherapist (n = 1), care coordinator (n = 3) and discharge planner (n = 1). Providers were between 27 to 69 years of age, 62.5% were female, and ten providers had practiced for five years or more in their current role. Of the 16 providers, 13 reported being ’very comfortable’ and 14 reported being ‘somewhat comfortable’ with using a computer and smartphone/tablet. Interviews were conducted between January 2021 and April 2021.

Generally, hospital and community providers experienced similar challenges while supporting hospital-to-home transitions for older adults during the COVID-19 pandemic. Providers often felt uncertain about available resources and felt that the expectations of the providers in the other setting were unclear or simply unrealistic within the constraints of the pandemic context. Overall, four interrelated themes were identified: 1) More tools, more time, more communication disruptions, 2) Discharge planning gets even more complex, 3) Changes in patient care mean significant changes for family caregivers, and 4) Technology was a good substitute, but not ideal (see Table 1 for the themes, subthemes, and quotes and Fig 1 for a visual summary of the themes and subthemes).

**Table 1:** Themes, subthemes and supporting quotes.

Theme	Subtheme and supporting quotes
***Theme 1*: *More tools*, *more time*, *more communication disruptions***	**Subtheme 1a Interprofessional communication**
	***P3*, *hospital provider***: “*Ordinarily we are accustomed to having our team rounds where we discuss progress that all the patients are making*, *rehab goals*, *and discharge planning issues*. *So we did that previously in a small room on the clinical unit and as a part of that we have kind of a visual tool on the television screen that tells us where the patient is with regard to their estimated length of stay in it helps make discharge decisions*. *So that the way it’s changed now is that we have moved the location of our interdisciplinary weekly team rounds to the dining hall which is just outside of our clinical unit*. *We are spaced out and we don’t have the visual tool that we ordinarily have that assists us with making discharge planning decisions and so I would say that’s probably the biggest thing that has changed from an interdisciplinary perspective…Without the visual tool it doesn’t give us the information that we need with regards to estimated length of day*. *I would say probably length of stays have increased because of the loss of that tool*.*”* ***P4*, *community provider***: “*There is people within the Family Health team that I don’t necessarily always connect with all the time*, *whether it’s the dietitian or the diabetes consultant*. *I just have no need to connect with them on regular basis but when I see them in the hallway*, *I may have a quick question for them*, *and they may have a quick question for me*. *Right*? *So*, *it’s the in-person presence that is helps building better relationships for us as professionals and consequently as a result for patients*. *Information sharing is probably better in person overall*. *The amount of information being shared*.”
	**Subtheme 1b Communication with patients**
	***P8*, *hospital provider***: *“During COVID*, *a lot of the assessments I still have to do what I can with the patient face-to-face*.*”* ***P16*, *community provider***: *“It’s hard to provide good care virtually to a patient who was admitted to the hospital with a complex problem that you were not involved with*. *And now you have to jump in virtually to pick up the slack*. *That’s very challenging*.*”*
	**Subtheme 1c Communication with caregivers**
	***P3*, *hospital provider***: “*We used to have more frequent interdisciplinary family meetings with caregivers and family members involved in that process*, *but we’ve and we would typically have that in a smaller room and there would be a number of people in the room at the same time*. *So*, *we haven’t been doing as many of those and when we do those they are improvised*, *fewer people*, *bigger space and probably fewer members of the interdisciplinary team involved*.*”* ***P12*, *hospital provider***: *“Communication with families has decreased quite a bit owing to the face that we don’t engage with them as much as we used to*. *That personal engagement is gone*. *Before*, *families would be present by the bedside more often… but now that probably (of them being around) is so much lower*, *so that ad hoc conversation gets much harder*. *So we end up calling the families*.*”*
***Theme 2*: *Discharge planning gets even more complex***	**Subtheme 2a Additional barriers to managing transitions from the hospital**
	***P6*, *hospital provider***: *“There used to be access to more services before COVID but now all of the services have changed*, *or they are completely on hold*. *So*, *for example we had a service that previously would bring patients home and on the way home they could stop at the pharmacy*, *they could stop at the grocery store*, *they could do all the errands you would need to do on your way home from the hospital but now*. *However*, *since COVID*, *they no longer make stops at the pharmacy or grocery store*. *So*, *it’s an extra piece to coordinate in terms of how are they going to get food on their first day home*? *How are they going to get their meds on their first day home*? *It added extra coordination*. *There are also other services that are completely cancelled…And then another challenge has been with COVID there seems to be a lack of PSW that is available*. *So*, *the team can make the recommendations in terms of what they recommend but it seems like in reality it’s much more difficult to actually secure as much PSW hours as compared to before I guess*.*”* ***P12*, *hospital provider***: *“Discharges back to home have been impacted only because services are hard to obtain*. *So many PSWs are unable to work that it’s hard to secure services*. *Patients are kept longer in the hospital until the supports can be found*.*”*
	**Subtheme 2b Discharge service restrictions and delays in the community**
	***P9*, *community provider***: *“If you look at the actual processes*, *not much has changed from hospital to home*. *However*, *because of COVID and the pandemic*, *there’s a lot of external factors that affect the care and service delivery*. *For example… because there was a fear of personal support workers coming into the home*, *a lot of families actually put services on hold in the beginning of the pandemic*. *Families were also very reluctant to taking patients home from the hospital*. *Also*, *because of the pandemic*, *a lot of the service workers were also taking a leave or taking a break… this would affect what the capacity is for services in home*. *So*, *then that would delay a discharge because we now don’t have people or personal support workers who can go in in time for the discharge from the hospital*.*”*
	**Subtheme 2c Additional work**
	***P2*, *hospital provider***: *“It is a little bit more difficult because of the visitor policies*. *It’s also a bit more time-consuming for us because it involves a lot of*, *with patient consent we either email caregivers or talk on the phone*, *but a lot of times before the families would naturally be with the patients but now*, *we have to do a lot more*.” ***P16*, *community provider***: “*(Caring for patients post discharge from the hospital is) very challenging*. *It was challenging before COVID*, *it’s a lot more challenging now*. *(For example)*, *because the family doesn’t have direct access to the patient*, *like being at the bedside*, *where before*, *there often was a family member there who (the doctors in the hospital) can talk directly to*. *But now that’s now there*, *so I’m getting a lot of calls from family members asking me what’s going on*.*”*
	**Subtheme 2d Barriers to care provision**
	***P2*, *hospital provider***: *“Sometimes we used to do home visits when patients were in hospital…We can’t do that anymore*. *So*, *we are just trusting what we think based off like pictures and what the family is saying and then we put the [de-identified homecare agency] in but if it was a complex discharge*, *we had the option of going in but that has changed*.*”* ***P9*, *community provider***: *“If you look at the actual processes*, *not much has changed from hospital to home*. *However*, *because of COVID and the pandemic*, *there’s a lot of external factors that affect the care and service delivery*. *For example… because there was a fear of personal support workers coming into the home*, *a lot of families actually put services on hold in the beginning of the pandemic*. *Families were also very reluctant to taking patients home from the hospital*.*”*
	**Subtheme 2e Increased homecare support**
	***P14*, *hospital provider***: “*(These new homecare programs) are more holistic*, *they allow for more personal support worker support*, *they allow for longer periods of time for people to be with patients when they returned (home)*. *So*, *these programs are really great*.*”*
***Theme 3*: *Changes in patient care mean significant changes for family caregivers***	**Subtheme 3a Difficult for caregivers/families to accept changes in patient’s status**
	***P10*, *hospital provider***: *“One thing I do hear from a lot of families*, *just overall on the pandemic is*, *because they haven’t seen their loved one*, *it’s hard for them to know really how the patient is doing*, *(versus being able to) eyeball the patient and really knowing*. *… The therapist will say*, *you know*, *for example*, *“Your dad needs help now*, *he would need someone to remind him to take his pills*.*” And they will go “Oh*, *he was taking his pills by himself before”*, *or ‘he didn’t need help going to the toilet*.*’ So they haven’t eyeballed the patient and that’s tough for them*. *For me*, *it’s making it transparent to them that the reality is when your loved one goes home now*, *maybe this is the new (normal)*.*”*
	**Subtheme 3b Additional caregiver stress and burden**
	***P9*, *community provider***: “*Transition from hospital-to-home is*, *it is a scary time…A lot of times patients decondition in hospital so now when they are weaker so when they come into the home there is always that fear of that you were in a place where you could have been exposed to COVID…I think there is also that fear of service workers coming into the home as well*. *Especially for complex patients who require that require additional PSW*. *So*, *you are having 2–3 people come in every day*. *So*, *a lot of families would like to limit the number of people coming in*. *So*, *I do have people request that it is the same people coming in and if they can’t*, *they have to manage*.*”*
***Theme 4*: *Technology was a good substitute but not ideal***	**Subtheme 4a Technology used to support transitions**
	***P12*, *hospital provider***: *“I think people [providers on the unit] are more comfortable doing a teleconference rather than Zoom because you’re unsure as to what technology access people have*. *I think we just end up doing a telephone number because everyone can do that*.*”* ***P1*, *community provider***: *“Most of the patients that have been discharged are older*. *We have the capacity to do video visits but that is really not the norm*. *I would say that is 10% of our visits that are virtual and 90% of them are phone call*.*”*
	**Subtheme 4b Concerns about technology**
	***P7*, *hospital provider***: “*The elderly may not be comfortable with technology… they may not be able to go online and look at something… I find that elderly people are not able to use technology… everyone generally speaking is good with technology but there are people that cannot really use computers or a smartphone and their day today and that can be challenging when you have an elderly patient*.*”* ***P9*, *community provider***: “*Seeing someone from a webcam and seeing someone in-person is completely different*. *I mean you can see someone’s emotions*. *You can see their face*. *It’s almost like seeing a picture*. *But when you are seeing someone in-person you can see how they move*, *where they are*. *It is little things that you can pick up on in-person rather than webcam…a lot of my patients are elderly*, *so they are more comfortable with a telephone rather than webcam*.”
	**Subtheme 4c Perceived opportunities for technology to support transitions**
	***P5*, *hospital provider***: *“Video could be a good option for…if you’re doing a home assessment to kind of have the team see how the patient’s bathroom is*. *Sometimes they take pictures*, *but I think video could be a good option and also demonstrating transfers to the caregiver*.*”* ***P4*, *community provider***: “*Communicating interprofessionally amongst the professional*, *whether it’s the hospital or in the community*, *I think there’s a huge*, *huge use for technology and that we haven’t*, *we’re still not using the things we could*. *So*, *the nurse in the community*, *we send the nurse to do something like wound care But*, *that report is not necessarily getting back to the doctor*. *They aren’t communicating with the doctor*. *Well*, *they are if there is someone like me in the middle making sure that communication can happens but I think there are ways we can improve communication*, *whether it’s through an e-health*, *connecting Ontario or something like that that everyone has access to*. *It’s helping to share information*.*”*

### Theme 1: More tools, more time, more communication disruptions

While more communication tools were used during the pandemic, communication took longer and was more disrupted. Table 2 outlines the transition-related communication disruptions that providers deemed essential during care transitions.

**Table 2:** Transition-related communication disruptions during the COVID-19 pandemic.

What communication was disrupted?	How was communication disrupted?	Communication sender/receiver?	What was the impact of disruption?
***During discharge planning in hospital***			
communication about patient’s status and needs	rounds without visual communication/tools	hospital interprofessional team	discharge planning not as productive
communication about patient’s care plans, status and needs	personal protective equipment made it difficult to communicate (lack of non-verbal cues, reduced sound)	hospital providers and patient	difficult for providers to assess patient’s level of function and plan for their care needs after discharge
communication about patient’s care plans, status and needs discharge instructions/resources	due to restricted visitor policies, communication occurred by email or phone	from hospital providers to caregivers	rapport/trust between caregiver and provider caregivers less prepared to assist patient providers unclear whether caregivers received/understood information additional administrative tasks and time to providers workload, direct reduced patient care time
referrals to community services	unclear whether resource/service was still available and referrals were received	from hospital to community providers	patients may not receive needed community resource/service
***After discharge home from hospital***			
7-day follow-up	due to restricted in-person visits providers had to selectively determine which patients had to be seen in hospital versus virtual care -patients and caregivers were not always willing to attend in-person visits	community provider and patient	greater risk of misdiagnosis patients had decreased care-seeking behaviour virtual care provided alternative to in-person visits and quicker follow-up
patients’ willingness to seeking care	patients and caregivers unwilling to seek in-person care when symptoms arise due to fearing getting COVID-19 from healthcare institution/provider	patient, hospital and/or community provider	patients may not identify adverse events and can lead to poor management of care needs and overall poor health status
community services/resources	patients and caregivers declining community services and resources	patient, caregiver, community providers	caregivers having to take on more responsibilities patient’s health can decline reduced opportunity to recognize adverse symptoms

#### Subtheme 1a interprofessional communication

Hospital providers explained that the pre-pandemic format of multidisciplinary rounds, used by the interprofessional healthcare team to collaboratively plan a patient’s discharge, was modified to adhere to physical distancing requirements. While these rounds occurred in-person prior to the pandemic, COVID-19 caused some units to conduct this using teleconferencing (i.e. a telephone call with more than two simultaneous participants). In contrast, others continued in person but were moved to a larger room to allow physical distancing. However, as P13 (hospital provider) described below, when rounds were conducted over teleconference, information tended to be more easily overlooked and less information was shared by the team.

"I would say daily rounds are not as productive as they were when we were all able to be in the same room. For a number of reasons. I think it’s just more difficult and challenging over teleconference. So, I think people are less likely to interrupt, or there are not so much miscommunications, but I think they take a little longer, number one. I think to be honest we glaze over things that we may have previously had gone over more because we find that rounds are more time-consuming doing them over teleconference."

Providers who participated in physically distanced in-person rounds reported communicating challenges with the team while wearing Personal Protective Equipment (PPE). Moreover, they did not have access to their usual visual planning tool due to moving to a different space. As a result of these changes, interprofessional communication around discharge planning was perceived as less productive during the pandemic.

In the community, P4, a community care coordinator who conducted hospital and home visits, and worked remotely prior to the pandemic, was less impacted by the shift to remote communication and care than those who were less accustomed to remote communication.

"One of the most interesting things when COVID hit, and everyone was sent home, so I’ve been doing this for 10 years already. So the next day, nothing changed for me. I work all over the place, you know? I joke I’m like a turtle, and I carry everything on my back. I can work wherever I am. So at the moment, I have been working more from home…From that perspective I think we were fairly technologically-advanced with laptops that had access to [Internet] and VPN. All of those things were very familiar for me which I don’t think were for others."

However, all community providers, including this care coordinator, felt that in-person interprofessional communication related to patient care was more comprehensive and productive. Additionally, due to technology being used more frequently to support remote communication, one community provider (P1) was concerned that communication with new medical residents regarding medical training was negatively impacted. For instance, P1 explained that it was much harder for residents to learn rapport-building skills and hone their observational assessment and diagnostic skills.

#### Subtheme 1b communication with patients

During the pandemic, hospital providers continued to communicate with patients in-person but experienced challenges while wearing PPE due to a lack of non-verbal communication (e.g. facial expressions) and reduced speech volume. As a result, communicating with patients took longer and increased the risk of misinterpretation than communication in the pre-pandemic context.

In contrast to hospital providers, most community providers interacted with patients over the phone as in-person visits were restricted to patients on a case-by-case basis. However, determining which patients required an in-person or remote visit simply based on verbal information shared by the patient or caregiver over the phone was challenging. Providers reported that patients preferred not to attend in-person appointments in some instances due to a fear of COVID-19 transmission from the healthcare institution. Concerning phone communication, providers were concerned that the lack of observational data increased the risk of misdiagnosis. Community providers indicated that phone interactions took much longer than in-person assessments as they were not trained or accustomed to assessing and diagnosing patients over the phone. Remote assessments took longer because providers had to ask patients or caregivers more questions to compensate for the lack of observational data.

#### Subtheme 1c communication with caregivers

During the pandemic, both hospital and community providers communicated with caregivers primarily through phone or email more often than they did pre-pandemic. The type of information that was shared with or sought from caregivers differed between hospital and community providers. Hospital providers communicated with caregivers to obtain necessary historical information and provided information about discharge plans (e.g. a patient’s baseline or current health and function, medication and resources/support available after hospital discharge). Community providers communicated with the patient and caregiver remotely at the same time. Community providers addressed and managed patient and caregiver concerns or needs after hospital discharge. Both hospital and community providers agreed that remote communication enabled them to share the necessary information with caregivers. However, they were concerned that remote communication negatively impacted the provider-caregiver relationship and caused caregivers to mistrust the information given to them by providers.

"I think a lot of families, they get it but I do also hear from the families that you know because they haven’t seen their loved one, it’s hard for them to even though we as professionals are saying ’oh you know, for example, your dad needs help now. Someone will need to remind him to take his pills, you know? But they will go ’oh he was taking his pills by himself before’ or ’they didn’t need help going to the toilet before‴

(P10, hospital provider).

### Theme 2: Discharge planning gets even more complex

#### Subtheme 2a additional barriers to managing transitions from the hospital

While hospital providers indicated that discharge processes were not overly different during the pandemic, they believed that discharge planning was "more complex" (P2, hospital provider) because there were additional discharge barriers to manage. The first barrier participants identified was that providers were uncertain about the availability of community resources and services and patient willingness to receive them. Providers explained that the availability of community resources and services had constantly changed during the pandemic. Hospital providers were uncertain about which community services they could refer patients to after discharge and were concerned that patients were falling through the cracks (i.e. not getting access to the community services they needed). P8 (a hospital provider) explained that this uncertainty was caused by less frequent communication between hospital and community organizations compared to pre-pandemic when fewer updates/communication between hospital and community staff about service availability was required because community services were more consistent. Another factor complicating discharge planning was that providers also had to consider alternative discharge supports and resources to recommend or refer patients because patients and caregivers were declining recommended community services due to a fear of COVID-19 transmission from community providers.

The second discharge barrier was that hospital providers found it harder to obtain the information they needed to plan discharges (e.g. patient’s baseline, resources available, etc.), mainly from patients who had language or communication difficulties. They explained that this information would be readily available from caregivers who were often at the patient’s bedside prior to the pandemic. P2 (a hospital provider) explained that they had to manage these additional discharge barriers while managing added pressure from the organization to quickly discharge patients as organizations attempted to create capacity to care for the overwhelming number of patients with COVID-19. Third, hospital providers explained that discharges were being delayed directly by COVID-19 in instances of an outbreak on the unit or if the patient’s household members had the virus.

"We are trying to get patients out of rehab as fast as possible…we are trying to offload patients from acute care so they are coming to us so that we are offloading from them, but we are also trying to get people home as fast as we can, safely. So that way we can keep taking people"

(P2, hospital provider).

Compounding this, providers explained that staffing shortages added to the stress and pressure they faced while facilitating transitions during the pandemic. They indicated that at the start of the pandemic, staffing shortages were caused by hospitals operating at overcapacity and sick calls from staff due to redeployment, illness or having to isolate after being exposed to the virus.

#### Subtheme 2b discharge service restrictions and delays in community care

Similar to hospital providers, community providers were concerned about patients having restricted or delayed access to recommended community services, which were limited by community staff shortages or patients refusing these services. Additionally, community providers expressed concerns about discharge services delays as they believed unreasonable expectations were imposed on them. For instance, P16 (a community provider) explained that it was unrealistic for hospital providers to expect community providers to schedule in-person follow-ups with patients within seven days of hospital discharge during the pandemic because of the limited capacity to schedule in-person appointments. Moreover, if patients were infected with the COVID-19 virus, patients were temporarily ineligible to receive medical testing/imaging, which further delayed discharge services in the community.

#### Subtheme 2c additional work

Hospital providers indicated that they had to take on additional tasks during the pandemic, which added to their existing overwhelming workload. For instance, prior to the pandemic, they explained discharge instructions to patients and caregivers simultaneously as both were present in person. However, during the pandemic, hospital providers indicated that they had to explain discharge instructions twice—once in-person to the patient and then again to caregivers over the phone or through email. Moreover, prior to the pandemic, providers were aware of which services were available; however, many services were temporarily closed or understaffed during the pandemic. As such, providers explained that they frequently contacted community organizations to confirm service availability during the pandemic. This additional work was perceived as problematic because it reduced time for direct patient care.

Similarly, community providers indicated that they were obliged to assume additional work during the pandemic. Due to patients being discharged from the hospital sooner than they would have been pre-pandemic, community providers explained that they had to care for patients who were sicker and required more medical intervention. Moreover, community providers indicated that patients and caregivers were contacting community providers more often with questions relating to their health and care while in the hospital. The increased communication with community providers resulted from caregivers having limited involvement in the patient’s hospital care compared to before the pandemic. Community providers felt limited in their responses, as they did not always have comprehensive details about the care provided in the hospital.

#### Subtheme 2d barriers to care provision

Hospital providers found that conducting assessments with patients (a vital part of care provision) during the pandemic was challenging for multiple reasons relating to infection control measures. Due to infection control measures, providers were restricted from taking materials (e.g. cognitive assessments) into the hospital rooms. In addition, patients were restricted from using the hospital therapy gym during a mandatory seven-day isolation period after arriving on the unit or if they tested positive for the COVID-19 virus, limiting patients’ functional progression. Finally, hospital providers explained that planning discharge for patients with complex care needs was more difficult due to organizational policies and infection control measures restricting home visits.

Community providers explained that some policies implemented during the pandemic to reduce the risk of virus transmission impacted care provision. For instance, these new policies directed community providers to deliver care virtually while restricting in-person care. However, providers expressed concerns with virtual care, including increased risks of misdiagnosing due to the inability to perform physical assessments. Many providers felt that telephone-based assessments failed to provide them with a comprehensive understanding of the patient’s condition. Most providers considered virtual care inferior to in-person.

#### Subtheme 2e: Increased homecare support

A hospital provider explained that some discharge concerns were alleviated with a new program designed to increase home support to a level that previously believed was impossible. The program aimed to support early discharges and create hospital capacity to manage the increased number of hospitalized patients. This program was beneficial in care transitions, allowing providers to discharge patients earlier as these new homecare programs could manage their care needs.

"We’ve seen different programs that are allowing the [de-identified homecare agency] to have different supports to do different programs that allow for patients to get out of the hospital with whatever means necessary, essentially. So being able to do enhanced programs or high-intensity supports at home. Like for rehab, physiotherapy and occupational therapy, being able to do home programs that allow patients to get out of the hospital with a higher level of support when maybe otherwise they wouldn’t be able to do that."

### Theme 3: Changes in patient care mean significant changes for family caregivers

#### Subtheme 3a difficult for caregivers/families to accept changes in patient’s status

Hospital providers perceived that caregivers and families had difficulty trusting and accepting information about changes to a patient’s baseline health and functional and physical status level because of restricted visitation. Restricted visitation was due to restricted visitor policies in the hospital and caregivers’ hesitancy to visit patients. Providers explained that caregivers might be unprepared to manage a patient’s care needs without a comprehensive understanding of a patient’s status after hospital discharge.

#### Subtheme 3b additional caregiver stress and burden

Hospital and community providers explained that transitions home from the hospital were even more stressful for caregivers during the pandemic for multiple reasons. First, caregivers were required to take on additional care activities after a patient returned home from the hospital if they declined community services or the services were unavailable. Second, hospital providers perceived caregivers were less prepared to manage a patient’s care after hospital discharge because caregivers had fewer opportunities to be involved in discharge planning due to limited hospital visitations. Third, hospital providers indicated that caregiver education/training, an essential part of the discharge process, primarily occurred through email or phone during the pandemic. Although providers noted that communication by telephone was helpful in some cases (e.g. caregivers not having to wait at the hospital to speak to a clinician), this format was less comprehensive as it did not allow hands-on demonstrations of discharge instructions.

### Theme 4: Technology was a good substitute, but not ideal

#### Subtheme 4a technology used to support transitions

Hospital and community providers used technology for different purposes during the pandemic. Hospital providers used faxes, electronic referrals, phone calls, videoconferencing and emails to communicate with other healthcare providers in the same or different organizations and gather/share information with caregivers and family members (e.g. during family conferences). Also, hospital providers would sometimes use the same medium that the patient was already using to communicate with their caregiver or family member (e.g. Facetime, WhatsApp, etc.). To streamline information sharing, P3 (a hospital provider) indicated he would try to schedule his patient visits to coincide with a time that patients were already communicating with their caregiver or family member: "What I started to do, and I think this is actually an enhancement in care is that I would actually go to the bedside and phone the family member from the bedside with the patient present and I think that has been working out ok."

Community providers indicated using various technologies during the pandemic, including one-way email, phone calls or sometimes videoconferencing. These technologies were used to connect with patients, caregivers, and other providers to assess, diagnose, and exchange information. Providers indicated that they often used one-way emails and telephones to communicate with patients or caregivers. They agreed that these mediums allowed them to continue delivering care, but they highlighted several concerns which impact the post-pandemic sustainability of these technologies in routine care.

#### Subtheme 4b concerns about technology

Hospital and community providers explained that they rarely used videoconferencing due to technical issues and concerns. Providers also assumed that older patients and caregivers might not be comfortable or have the resources and skills to use videoconference. Providers also indicated that a lack of comfort with videoconferencing technologies and logistical issues (e.g. work laptops not being equipped with cameras) were barriers to using videoconference. P4 (a community provider) expressed concerns that remote visits would not allow providers to identify essential issues in the way that in-person visits would:

"What is missing now is the in-person visits to client’s homes. There is no replacement to that. To physically see someone how someone moves around, to physically see their environment, you know? Everything. The smell, the feel, the walking up to the building, all those issues. Walking through the door, are they able to get their wheelchair over the door threshold? You know what I mean? There is all these things you wouldn’t notice even in a virtual environment that I think is definitely missing at the moment."

Another concern was that using technology with patients who had cognitive, visual or hearing impairments was challenging. However, providers rarely mentioned privacy concerns as many providers trusted their organization’s security measures. Finally, providers indicated that incorporating technology into their workload added another task.

#### Subtheme 4c perceived opportunities for technology to support transitions

Providers believed that some aspects of virtual care (e.g. email and phone communication with patients and caregivers) might continue beyond the pandemic. While face-to-face was generally preferred, providers believed that email and phone could supplement or, in some cases, be an alternative to in-person communication. For instance, P11 (hospital provider) indicated that remote communication was beneficial for caregivers as it minimized travel burden and the amount of time they spent in waiting rooms. P1 (a community provider) indicated that phone calls might be appropriate for following up with patients after hospital discharge who have transportation barriers or other conditions limiting their ability to participate in an in-person encounter. Given the pandemic context, virtual care was considered an adequate substitute for in-person communication but not ideal.

## Discussion

This study describes hospital and community providers’ experiences facilitating hospital-to-home transitions during the COVID-19 pandemic. Generally, providers perceived these transitions as more stressful and challenging to plan and coordinate compared to the pre-pandemic context. The finding resonates with previous research, which has acknowledged high levels of provider stress as a critical issue during the COVID-19 pandemic [66, 67]. Based on our study findings, reducing uncertainty and unrealistic expectations imposed on providers could reduce provider stress and improve transitions during and after the COVID-19 pandemic.

### Communication in transitions during the pandemic

The discharge barriers described by providers were often related to poor communication, indicating that several discharge barriers experienced during the pandemic were caused by poor communication. Although poor communication between providers, patients and caregivers during transitions has been a concern before the pandemic [68], the pandemic context brings new insights into what communication providers deem essential during care transitions (see Table 2 for a list of transition-related communication disruptions). For instance, previous research emphasized the role of verbal communication in the discharge process [68]. Our study extends this finding by highlighting the value of non-verbal communication between patients, caregivers, and providers and among providers equally as crucial during the transition process. In line with previous findings [69], our findings indicated that non-verbal communication was more of a concern for hospital providers who had no prior relationship with patients and caregivers but less of a concern for community providers who had pre-existing longstanding relationships with patients.

### Discharge facilitators and concerns during the pandemic

Our findings revealed that additional community resources enabled providers to discharge patients earlier than pre-pandemic. This highlights the possibility of earlier discharges through programs designed to support medically complex patients in the community. However, the uncertainty about the duration and sustainability of new community programs may impact their use and usefulness [70, 71]. It is imperative now, as experts believe that patients have been "storing up" health concerns during the pandemic, and there may be an increase in demand for healthcare when these concerns become urgent and more substantial [72, 73].

### Technology in transitions during the pandemic

Providers had identified concerns and barriers to using technology to support transitions during the pandemic that must be addressed in future work. Telehealth enabled care delivery while minimizing the risk of COVID-19 transmission between patients, caregivers and providers. However, providers indicated that telehealth was inferior to in-person care because it was not ideal in all situations. For example, providers found that telephone follow-ups increased convenience and allowed them to reach those patients they previously could not reach (e.g. due to transportation issues and not feeling up for an in-person visit). However, they were concerned that remote assessments could lead to misdiagnosing, a concern previously noted as the efficacy of telediagnosis has not been validated yet for acute conditions [69, 74]. Despite providers emphasizing the need for observational data and non-verbal communication during transitions, providers rarely used videoconferencing. Consistent with previous studies [75], barriers to the use of videoconferencing were experienced by providers in the hospital and community, including a lack of facility equipment (e.g. webcam, laptop), the time needed to set up telehealth meetings along with a lack of provider and patient comfort with the technology, technical issues encountered in previous use, and equity concerns related to a fear of further disadvantaging underserved populations. Provider concerns, including difficulties with using such technology with elderly patients and those with visual or hearing impairment, should be accounted for in technology design to support use and uptake by providers. However, given the numerous concerns highlighted by providers, it remains unclear whether the future of transitional care will become increasingly digital.

Despite concerns related to technology use, providers emphasized that the value and benefits of technology cannot be ignored. The pandemic has highlighted the value of communication technology in healthcare. The lessons learned highlight critical areas of concern related to technology that could be addressed in future technology interventions supporting transitional care. For instance, our study revealed that when collaboratively creating discharge plans, providers perceived communication was more productive in-person and when visual tools were available. Information technology will need to support non-verbal communication and observational data to improve communication and patient and caregiver/family outcomes during the discharge process. Future research should also explore how non-verbal communication supports care transitions and platform preferences. Technology played an essential role in delivering care in the hospital and community, despite the numerous pandemic-related changes. Providers cited situations where technology streamlined processes and reduced caregiver burden (e.g. caregiver not needing to wait around to see the physician to get an update when this can happen over the telephone). In line with previous findings, our findings suggest that the use of telehealth after the pandemic may be helpful as an adjunctive or replacement to traditional community care only in some situations and in its current form, not a replacement [76, 77].

The COVID-19 pandemic has highlighted the power of healthcare policies to rapidly change how hospital and community organizations function [72]. For instance, in response to the new healthcare policies during the COVID-19 pandemic, in March 2020, primary care telehealth visits in the United States increased by 154% compared to the same time during the previous year [76]. Healthcare policies are needed to achieve large-scale changes, including the adoption of technological solutions [78]. Based on lessons learned from the pandemic context, continuing telehealth and adopting future health system innovations requires ongoing support from healthcare and organizational policies, clear guidelines for use, and further improvements to the technology design to better fit within the provider’s workflows.

### Limitations and strengths

The following limitations should be considered in the interpretation of these results. First, most participants completed a telephone interview rather than a video interview, which inhibited our ability to capture non-verbal cues and increased the risk of misunderstandings in the content they shared [79] and manage audio-recording data and video recording data. Second, this study reflects the perspectives and experiences of particular providers who worked in a publicly funded healthcare system and worked in urban settings. Their perspectives and experiences may differ from that of other clinicians. Third, to minimize the provider burden from study participation, we attempted to keep interviews with providers short. However, that may have reduced the depth of the conversations. Fourth, although we did not reach our target sample size (n = 20–30), we believe that the sample size was enough to achieve data sufficiency (i.e. “sufficient depth of understanding has been achieved in relation to emergent theoretical categories”), as perceived by our research team members [80]. Another weakness is the lack of patient/caregiver perspective. We had attempted to include patients and caregivers. However, this part of the project became unfeasible due to low sample sizes resulting from challenges individuals faced when transitioning home in a pandemic environment.

The team-based rapid qualitative methodology used within this study was a strength. This methodology integrated a multidisciplinary team-based analysis, which enhanced rigour and analytic depth [59]. Collecting and analyzing data concurrently and shortly after increases immersion in the data and the richness of analytic interpretations [59]. Moreover, another strength was examining the experiences of hospital providers at two distinct healthcare organizations, which improves the transferability of findings.

## Conclusion

In conclusion, healthcare providers in the hospital and community faced similar challenges while facilitating transitions during the COVID-19 pandemic. While technology was used to facilitate transitions during the pandemic, some concerns reduced utility and perceptions that the digital uptake would be sustained over time. Effectively sustaining positive changes beyond the COVID-19 pandemic may be possible by implementing practices that support sustainability, including routinizing and institutionalizing (e.g. creating a fit between the change, work, internal structures, organizational context and institutional requirements) [81]. However, as highlighted by Côté-Boileau & colleagues, more research is needed on how to support and operationalize the spread, sustainability and scale of healthcare innovations [81]. The opportunities identified for the use of technology to support transitions during the pandemic suggest that there may be a role for technology within transitions. Technology, such as email, tele- and video-conference, may also have the potential to address the transition-related communication challenges confronted by providers before the COVID-19 pandemic. However, more research is needed to determine where technology optimally sits in the transition workflow.

## Supporting information

S1 Appendix Interview guide. (DOCX)
