# Supplementary material for: A qualitative study of hospital and community providers’ experiences with digitalization to facilitate hospital-to-home transitions during the COVID-19 pandemic
Source: PLoS One. 2022 Aug 18;17(8):e0272224. doi: 10.1371/journal.pone.0272224 (PMC9387844; doi:10.1371/journal.pone.0272224)
Supplement: S1 Appendix — (DOCX) [file pone.0272224.s001.docx]

1. To start off, please tell me a bit about your role at [hospital/practice].

2. Next, I want to talk a bit about discharging patients from the hospital [primary care providers: what it’s like caring for a patient post-discharge]. Since COVID-19 there have been many changes in the way care has been provided in healthcare, can you tell me about what the discharge process looks like today and how the discharge process is different from pre-COVID?

-How has information sharing/communication with patients, caregivers and other providers changed? Clarify how ppl define communication; what are you looking for?

-What is going well?

-What is really challenging?

3. How has the COVID-19 pandemic made care transitions from hospital to home easier or harder?

4. How do you use technology as part of the discharge process if at all? Technology can be anything like fax, telephone, video call.

a. What technology, what information, devices required, training is needed?

b. How does this differ from what you are doing before COVID-19? (were you using this technology)?

-Do you have an example of when technology really helped the discharge process? (probe on: What is the technology? What information does it collect/transmit? Was training required? Any new devices used? What is the value of it for you/your patients/their family?

-Do you have an example of when you experienced challenges with technology to support discharging patients? (additional probes on: patient challenges in accessing and adopting and whether different groups of patients were able to uptake the technology differently; whether any groups were excluded from using technology and why that was the case)

-If you’re not using much technology, why do you think that is? And, what role do you think technology could play?

-Does anything worry you about using technology to help with discharge?

5. Do you give a choice of type of tech?

6. Do you think tech use will persist beyond the pandemic?

7. Is there anything else about the discharge process, technology, or the changing environment due to COVID-19 that you’d like to share, that we have not already talked about?
